# Selective sweeps and genetic lineages of *Plasmodium falciparum* multi-drug resistance (*pfmdr1*) gene in Kenya

**DOI:** 10.1186/s12936-018-2534-8

**Published:** 2018-10-30

**Authors:** Peninah Muiruri, Denis W. Juma, Luicer A. Ingasia, Lorna J. Chebon, Benjamin Opot, Bidii S. Ngalah, Jelagat Cheruiyot, Ben Andagalu, Hoseah M. Akala, Venny C. S. Nyambati, Joseph K. Ng’ang’a, Edwin Kamau

**Affiliations:** 1 Global Emerging Infections Surveillance Program, United States Army Medical Research Directorate-Africa, Kenya Medical Research Institute, P.O. Box 54, 40100 Kisumu, Kenya; 2 Department of Biochemistry, School of Biomedical Sciences, Jomo Kenyatta University of Agriculture and Technology, P.O. Box 62000, 00200 Nairobi, Kenya; 3 Walter Reed National Military Medical Center, Bethesda, Maryland USA

**Keywords:** Soft selective sweeps, Genetic lineages, Artemisinin resistance

## Abstract

**Background:**

There are concerns that resistance to artemisinin-based combination therapy might emerge in Kenya and sub-Saharan Africa (SSA) in the same pattern as was with chloroquine and sulfadoxine–pyrimethamine. Single nucleotide polymorphisms (SNPs) in critical alleles of *pfmdr1* gene have been associated with resistance to artemisinin and its partner drugs. Microsatellite analysis of loci flanking genes associated with anti-malarial drug resistance has been used in defining the geographic origins, dissemination of resistant parasites and identifying regions in the genome that have been under selection.

**Methods:**

This study set out to investigate evidence of selective sweep and genetic lineages in *pfmdr1* genotypes associated with the use of artemether–lumefantrine (AL), as the first-line treatment in Kenya. Parasites (n = 252) from different regions in Kenya were assayed for SNPs at codons 86, 184 and 1246 and typed for 7 neutral microsatellites and 13 microsatellites loci flanking (± 99 kb) *pfmdr1* in *Plasmodium falciparum* infections.

**Results:**

The data showed differential site and region specific prevalence of SNPs associated with drug resistance in the *pfmdr1* gene. The prevalence of *pfmdr1* N86, 184F, and D1246 in western Kenya (Kisumu, Kericho and Kisii) compared to the coast of Kenya (Malindi) was 92.9% vs. 66.7%, 53.5% vs. to 24.2% and 96% vs. to 87.9%, respectively. The NFD haplotype which is consistent with AL selection was at 51% in western Kenya compared to 25% in coastal Kenya.

**Conclusion:**

Selection pressures were observed to be different in different regions of Kenya, especially the western region compared to the coastal region. The data showed independent genetic lineages for all the *pfmdr1* alleles. The evidence of soft sweeps in *pfmdr1* observed varied in direction from one region to another. This is challenging for malaria control programs in SSA which clearly indicate effective malaria control policies should be based on the region and not at a country wide level.

**Electronic supplementary material:**

The online version of this article (10.1186/s12936-018-2534-8) contains supplementary material, which is available to authorized users.

## Background

Regardless of the emergence and spread of resistance to artemisinin-based combination therapy (ACT) in Southeast Asia (SEA) [1–5], ACT is still widely used as the first-line treatment for uncomplicated malaria worldwide [5] and has maintained high efficacy in sub-Saharan Africa (SSA) with fast clearance rates [5, 6]. SEA is historically considered to be major foci of anti-malarial drug resistance, where resistance to drugs such as chloroquine and sulfadoxine–pyrimethamine (SP) may have originated before eventually spreading to Africa [7, 8]. There is now heightened concern that resistance to ACT in SEA might follow the same pattern in expansion globally as previously did for chloroquine and SP [9]. With this in mind, routine monitoring of the therapeutic efficacy of ACT is critical in detecting early changes in *Plasmodium falciparum* sensitivity to anti-malarial drugs, and deemed necessary for timely enactment of changes to treatment policy [10]. Indeed, there is concerted effort to scale-up monitoring therapeutic efficacy of ACT in SEA [1, 2, 10–12], SSA [13], and the rest of the world [4].

Artemether–lumefantrine (AL) is the most commonly used ACT for the treatment of uncomplicated *P. falciparum* malaria worldwide [14]. AL was introduced as the first-line treatment for uncomplicated malaria in Kenya in 2006 due to widespread resistance to chloroquine and SP, in 1998 and 2006 respectively [15–17]. ACT has maintained adequate clinical and parasitological response (ACPR) in Kenya, with a recent study reporting more than 97% ACPR for AL and dihydroartemisinin–piperaquine (DP) in treatment of uncomplicated falciparum malaria in western Kenya [18]. However, studies have shown AL selects for single nucleotide polymorphisms (SNPs) in the *P. falciparum* chloroquine resistance transporter gene (*pfcrt*) and the *P. falciparum* multidrug resistance gene 1 (*pfmdr1*) in recurring parasites [19–24]. The genotypes associated with recurrent infections are K76 in *pfcrt*, and N86, 184F and D1246 (NFD) in *pfmdr1*. Reduced susceptibility to lumefantrine and mefloquine has also been linked to NFD and increase in *pfmdr1* copy numbers [25–27]. Chloroquine resistance is associated with *pfcrt* 76T [28], and modulated by *pfmdr1* 86Y, Y184 and 1246Y (YYY) [29]. The *pfcrt* 76 and *pfmdr1* 86 alleles are the most important indicators of chloroquine susceptibility [30]. Longitudinal studies have shown the prevalence of *pfcrt* 76T and *pfmdr1* 86Y reached over 90% in western and coastal regions of Kenya before the introduction of ACT, and reversed to the sensitive genotypes with the withdrawal of chloroquine and the introduction of AL [31–34]. This reversal to sensitive genotype in Kenya can be attributed to the release of chloroquine drug pressure and the introduction of lumefantrine drug pressure. Recent studies have suggested changes in the prevalence of these alleles can be a sensitive indicator of selection of parasite populations by AL which can be used to signal early reduced susceptibility [30, 35].

Kenya has a wide variation in malaria prevalence, with some regions free of malaria to those with more than 40% endemicity [36, 37]. Most regions in western Kenya are endemic lowland with high stable transmission whereas the highlands are characterized by unstable and high transmission variability which results in epidemics during periods of suitable climatic conditions [36]. To effectively monitor the emergence and spread of resistance to ACT, it is important not only to monitor the prevalence of these alleles (*pfcrt* K76, *pfmdr*1 N86, 184F and D1246), but also to monitor their origin and spread. Information on the evolutionary dynamics resulting in selection of these alleles in different parts of the country with different transmission intensities and different drug resistant profiles is important in guiding strategies to control, and prevent the emergence and spread of resistance to AL.

Microsatellites are important genetic markers used to identify regions in the genome that are under selection [38]. Genetic hitchhiking is driven by the selection process which results in reduction of heterozygosity at both the selected locus and neutral flanking microsatellite loci [39]. When the mutation eventually gets fixed in a population due to continuous selection, sequence diversity is reduced around the selected locus leading to selective sweeps [38].

Characterization of *P. falciparum* parasite genetic backgrounds using microsatellite loci flanking genes associated with resistance to chloroquine and SP was critical in defining the geographic origins and dissemination of chloroquine and SP resistant parasites. It has been reported that these resistant parasites originated in a few places before eventually spreading to the rest of the world [38, 40–43]. More recently, characterization of microsatellite loci flanking *pfmdr1* gene were used to provide comprehensive data on the distribution of alleles in this gene and the pattern of selective sweeps in four sites in Cambodia. These sites had different levels of transmission and drug resistance profiles [38]. The study established that *pfmdr1* 184F mutant allele was under selection in this parasite population whereas copy number variation of *pfmdr1* gene occurred on multiple genetic backgrounds. Given the importance of polymorphisms in *pfmdr1* gene in response to ACT, it is warranted to investigate the selective sweep and genetic lineages of *pfmdr1* alleles in SSA. This study set out to investigate evidence of selective sweep and genetic lineages in *pfmdr1* genotypes associated with AL treatment in Kenya. Microsatellite loci flanking *pfmdr1* gene in parasite population from different regions of Kenya with different malaria transmission intensities were characterized.

## Methods

### Sample collection

**Fig. 1:**
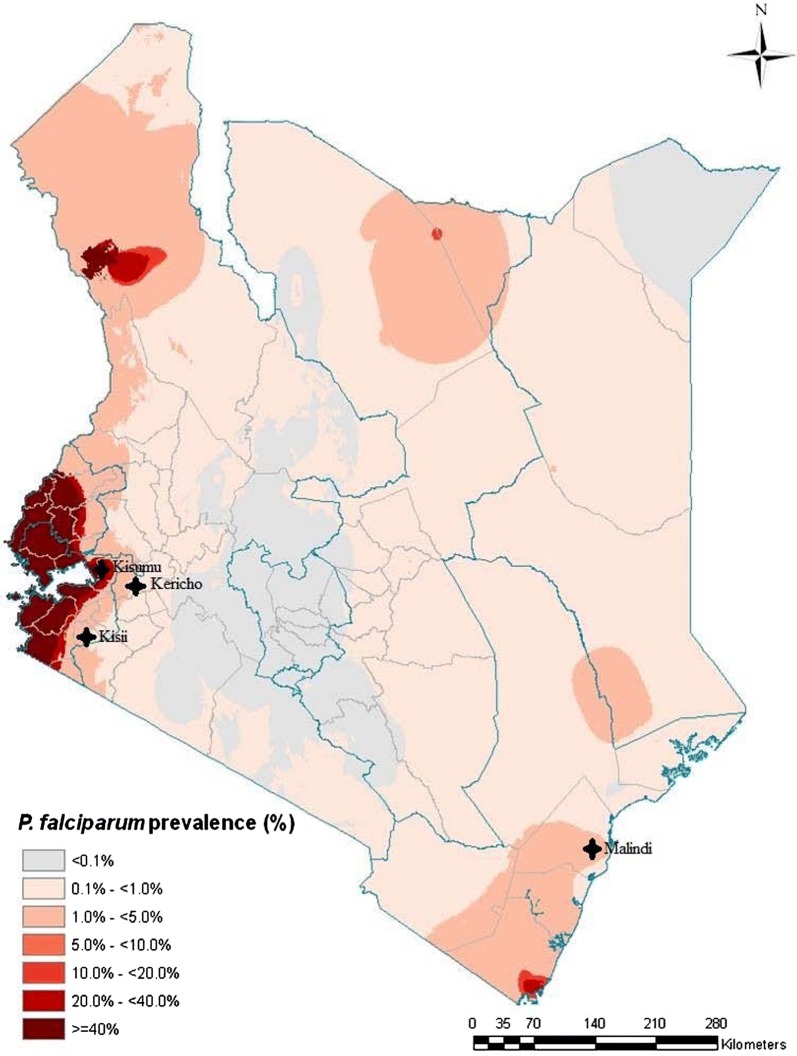
A map of Kenya showing the four sites from which samples were collected. These sites were chosen to cover different malaria ecological zones of Kenya with different transmission rates. The areas covered include the endemic lake (Kisumu) and coastal regions (Malindi; risk class equal to or above 20%), and the epidemic-prone highland region (Kisii and Kericho; risk class 5 to less than 20%) (From Noor et al. [37])

Whole blood samples from patients presenting with *P. falciparum* uncomplicated malaria were collected during the years 2012 and 2013 from four sites across Kenya (Kisumu, Kisii, Kericho and Malindi), 6–7 years after the introduction of AL as the first-line treatment for uncomplicated malaria (Fig. 1). Study participants were from either gender, aged between 6 months and 65 years with uncomplicated malaria presenting at the district hospitals in the above named regions. They were not on any anti-malarial treatment when they registered to the study (day 0 samples). Patients with complicated malaria, children below the age of 6 months, adults above 65 years, and those who were under treatment prior to enrollment to the study were excluded.

### *Plasmodium falciparum* DNA extraction and SNP genotyping

Parasite genomic DNA of 252 day 0 samples was extracted from 200 µL whole blood using QIAamp Blood DNA mini kit (Qiagen, Valencia, CA, USA) according to the manufacturer’s instructions. The extracted DNA was properly stored for the subsequent assays.

The parasite genomic DNA extracted from the samples was genotyped for key codons associated with drug resistance in the *pfmdr1* gene at codons 86, 184, and 1246 through direct sequencing. Previously described methods [44] were used for amplification and sequencing of two fragments of the gene (86–184, and 1034–1246). Sequencing was done on a 3500 Genetic Analyzer (Applied Biosystems, Carlsbad, CA, USA) platform.

### Typing of microsatellites, allele scoring and assessment of multiple infections

The parasite genomic DNA was used as template for amplification of 20 microsatellites loci which included 7 neutral microsatellites located on chromosome 2 and 3; and 13 microsatellites flanking downstream and upstream of the *pfmdr1* gene located on chromosome 5. The neutral microsatellites are used to assess natural variation in a genome that might be occurring in a population because they are not linked to the gene under selection [45]. Therefore, the neutral microsatellites can be used as a reference for selection in comparison with the microsatellites flanking the genes [SNPs] under selection. The *H*_*e*_ of the neutral microsatellites was compared to the *H*_*e*_ of the microsatellites flanking the *pfmdr1* gene to assess any evidence of selection using GenAlEx, as described by Peakall and Smouse in 2012 [46]. The neutral microsatellites were used to assess the clonality of each sample and included 3 loci on chromosome 2, (C2M29/319 kb, C2M34/313 kb, and C2M33/302 kb) and 4 loci on chromosome 3 (C3M40/335 kb, C3M88/363 kb, C3M69/383 kb and C3M39/429 kb) [45] spanning 94 kb. The primers and cycling conditions were adapted from previous studies [38, 47, 48]. The 13 microsatellites loci flanking *pfmdr1* gene are on positions − 99, − 54, − 30, − 9.3, − 4.2, − 3.3 and − 1.4 kb upstream and 0.16, 0.45, 3.6, 9.1, 23, and 89 kb downstream on chromosome 5 [45]. The PCR products were separated by electrophoresis on polyacrylamide gel with Genescan-500 LIZ labelled size standard on the 3500 Genetic Analyzer (Applied Biosystems, Carlsbad, CA, USA).

Allele scoring for all microsatellites was done using GeneMapper software, version 4.1 (Applied Biosystems, Foster City, CA, USA). The software automatically measures allele lengths and quantifies peak heights. Multiple alleles per locus were scored if electrophoretic peaks corresponding to minor alleles were ≥ 33% the predominant allele in the isolate and the peak height is > 200 rfu (fluorescence units) as previously described [49]. Predominant allele was defined as the allele with the highest peak height in the electropherograms. Peaks below 33% of the predominant peak and below 200 rfu were considered as poor quality and/or background noise. Since Kenya is a high transmission region, most of the samples had multiple alleles at any given loci. Parasite haplotypes cannot be determined when 2 or more clones are present and are usually removed from analysis. However, in a previous study, to present a population-based perspective, data from multiply infected parasites was retained for analysis where appropriate [50]. In this study, multiply infected parasites were retained but only the predominant alleles were used for allele frequency calculation and multi-locus microsatellite haplotype construction. According to Anderson et al. this method gives unbiased estimation of allele frequencies within a population assuming the composition of PCR products is representative of the composition of templates [50].

### Estimating expected heterozygosity (*H*_*e*_) and genetic differentiation (F*st*)

The genetic variation at each locus was measured as expected heterozygosity (*H*_*e*_). The formula for calculating the *H*_*e*_ is

$$ H_{e} = \left[ {n/\left( {n - 1} \right)} \right]\left[ {1 - \sum pi^{2} } \right] $$

where n is the number of isolates genotyped and pi is the frequency of the *ith* allele. This was estimated using GenAlEx v2.2. For the genetic differentiation (Wright’s fixation index, F*st*), values at selected loci are expected to be exceptionally low or high compared with loci that are not under the influence of any selection [51]. Subsequently *H*_*e*_ and F_ST_ were estimated at the 7 neutral microsatellite loci and the 13 microsatellite loci flanking the *pfmdr*1 gene to explore evidence of selection occurring at the gene. The *H*_*e*_ and F*st* at all the microsatellite loci were estimated using GenAlEx version 6.501 described by Peakall and Smouse [46].

### Constructing multi-locus microsatellite haplotypes and genetic lineages

The 8 closest loci (± 9 kb) around *pfmdr*1 (− 9.3, − 4.2, − 3.3, 1.4, 0.16, 0.45, 3.6 and 9.1 kb) were used for grouping the isolates or parasites into haplotypes using NETWORK software version 4.6.1.3. The use of the 8 closest loci around *pfmdr1* (as opposed to using all the 13 loci analysed) was important to allow us to compare this data with previously published data [44]. To define the genetic lineages of the resistant alleles in Kenya, we constructed the median-joining networks using NETWORK software as well. This enabled us to visualize the relationships among different alleles of the gene. Any locus that failed to amplify was assigned a null value (99) for the purposes of analysis.

### Statistical analysis

The mean He values were compared by using a Mann–Whitney U statistics and Chi square implemented in the statistical package GraphPad Prism (San Diego, CA, USA). A P value of ≤ 0.05 was considered statistically significant.

## Results

### Prevalence of polymorphism in *pfmdr1* codons 86, 184 and 1246

Of 252 isolates analysed, 94% (n = 236) were successfully genotyped in at least one of the codons and 73% (n = 185) of these had data obtained in all the three codons. Of the 236 isolates, 56% (n = 132) had single (pure) genotype in each of the codon where the sequence electropherograms showed single peaks. The prevalence of wild type alleles at *pfmdr1* 86, 184 and 1246 were 86.4%, 53.8% and 93.9%, respectively. Table 1 shows the prevalence of polymorphisms at *pfmdr1* 86, 184 and 1246 per study site. Kisumu had the largest number of samples analysed whereas Kericho had the least. The prevalence of the wild type alleles in *pfmdr1* 86 and 1246 were both highest in Kericho at 100% and lowest in Malindi at 70% and 89.5%, respectively. The prevalence of the mutant allele in *pfmdr1*184 was lowest in Malindi (27.8%).

**Table 1:** Prevalence of *pfmdr1* codons per study site

Region	Codon	N86Y	Y184F	D1246Y
		% (n)	% (n)	% (n)
Kisumu	Wild	96.7 (88)	47.5 (28)	93 (66)
	Mutant	3.3 (3)	52.5 (31)	7 (5)
Kisii	Wild	91.8 (56)	55.8 (29)	95.6 (43)
	Mutant	8.2 (5)	44.2 (23)	4.4 (2)
Kericho	Wild	100 (28)	38.9 (7)	100 (28)
	Mutant	0 (0)	61.1 (11)	0 (0)
Malindi	Wild	70 (28)	72.2 (26)	89.5 (34)
	Mutant	30 (12)	27.8 (10)	10.5 (4)

The number of isolates that were successfully genotyped at each codon from each of the study site. As an example: Kisumu N86Y codon had the highest number of successfully genotyped codons (total 91, wild type 88 and mutant 3) whereas Kericho Y184F had the lowest number of successfully genotyped codons (total 18, wild type 7 and mutant 11)

### Prevalence of haplotypes at *pfmdr1* codons 86, 184 and 1246

**Fig. 2:**
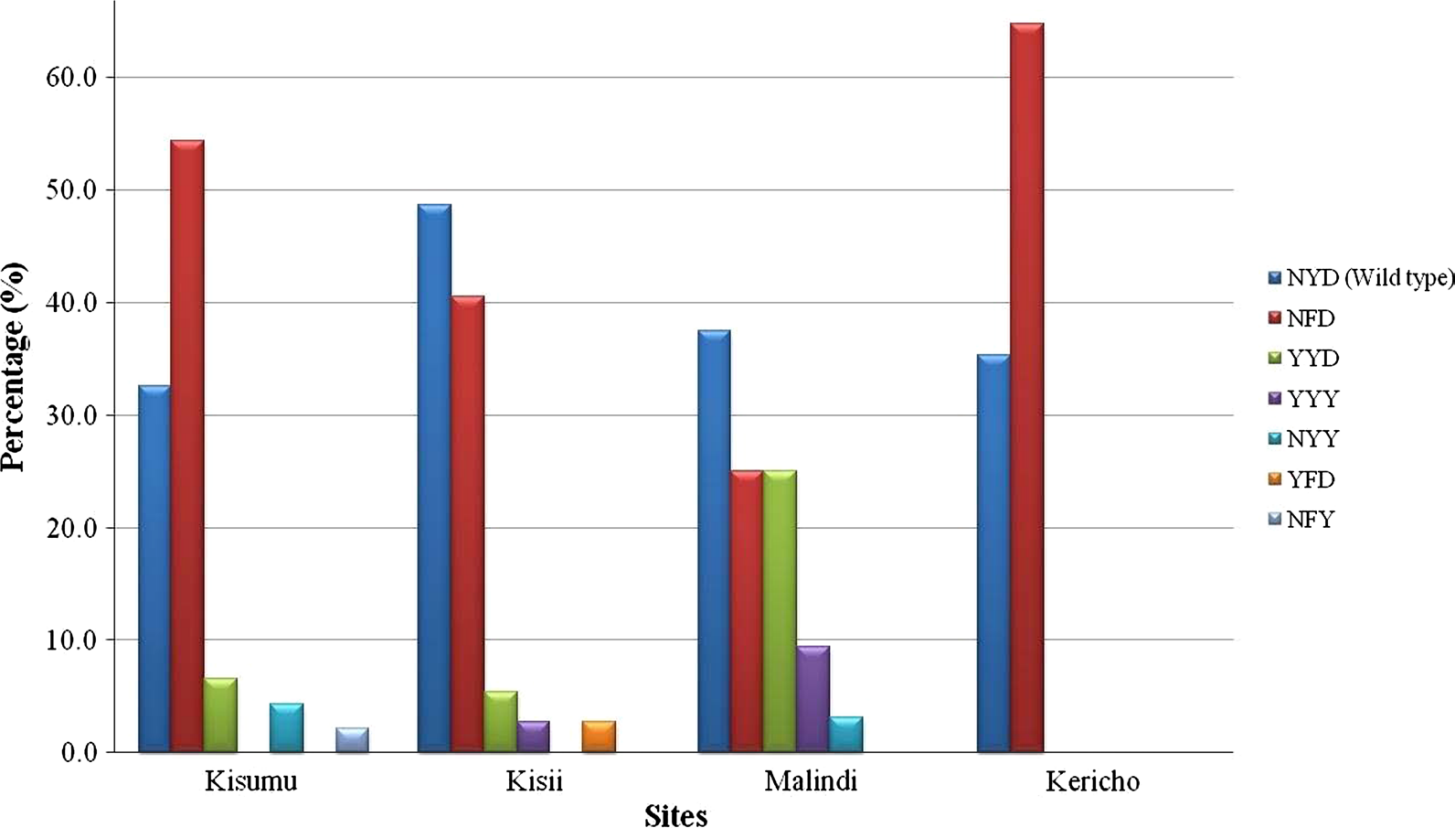
A graphical representation of Pfmdr1 SNP haplotypes prevalence per site. This shows the prevalence of Pfmdr1 codon 86, 184 and 1246 haplotypes between the different locations

For haplotype construction, only the 132 pure genotypes containing one allele per locus were analysed which were distributed as follows for each site: Kericho (17), Kisumu (46), Kisii (37), and Malindi (32). The most prevalent haplotypes among all the samples were N86, 184F and D1246 (NFD) at 44.7% (n = 59) and N86, Y184 and D1246 (NYD) at 38.6% (n = 51). Other haplotypes present were 86Y, Y184, D1246 (YYD) at 9.8% (n = 13); 86Y, Y184, 1246Y (YYY) at 3.0% (n = 4); N86, Y184, 1246Y (NYY) at 2.3% (n = 3); 86Y, 184F, D1246 (YFD) and N86, 184F, 1246Y (NFY) at 0.8% (n = 1) each. Regionally, NFD was the most prevalent haplotype in Kisumu and Kericho field sites whereas NYD was the most prevalent in Kisii and Malindi. Of the 13 parasites carrying YYD haplotype, 8 (61%) were found in parasites from Malindi and none in Kericho (Fig. 2).

### Evidence of selection in *pfmdr1* gene

Genetic hitchhiking of flanking *pfmdr1* loci resulting from the emergence of mutant alleles at *pfmdr1* codons 86, 184 and 1246 was studied. Out of the 236 samples analysed, only a few samples (16) were single clones based on having one allele in each of the 7 neutral microsatellites. This necessitated the use of the predominant allele per locus as described in the methods section in order to have sufficient number of samples for analysis. Further, of the 13 microsatellites flanking downstream and upstream of the *pfmdr1* gene that were assayed, only the 8 closest loci were used for the final analysis as previously described [44]. There was significantly less genetic diversity in the 8 microsatellite loci surrounding *pfmdr1* than was found in the neutral loci; the mean *H*_*e*_ (± standard deviation [SD]) surrounding *pfmdr1* [0.898 (0.036)] was 6% lower than the neutral loci [0.961 (0.075); *P* < 0.0014]. The *H*_*e*_ from the parasite populations from the four different field sites (Kisumu, Kericho, Kisii and Malindi) was then compared. The difference in the mean *H*_*e*_ across sites was small and did not reach statistical difference; Kisumu had the highest mean *H*_*e*_ [0.888 (0.033)] whereas Kericho had the lowest [0.845 (0.048); *P* = 0.083)].

To investigate the role of mutations in driving selective sweep, *H*_*e*_ of the 8 microsatellite loci around the different *pfmdr1* haplotypes was compared. The level of *H*_*e*_ was estimated around NYD, NFD and YYD. Due to small sample size, YYY (n = 3) and NYY (n = 2) were not considered in the analysis. In the overall parasite population, the reduction in *H*_*e*_ of the 8 loci surrounding *pfmdr1* was slightly more pronounced in parasites carrying mutant alleles compared to the wild type alleles. The difference in mean *H*_*e*_ between NYD [0.893 (0.038)] and NFD [0.876 (0.047]) haplotypes did not reach statistical difference [*P* = 0.51]. However, the mean *H*_*e*_ surrounding YYD [0.805 (0.055)] was 9% lower than NYD [0.893 (0.038)] reaching a statistical difference [*P* = 0.0074].

For site specific analyses, the mean *H*_*e*_ of the 8 microsatellite loci around NYD and NFD haplotypes in parasite populations from Kisumu, Kericho and Kisii were compared. For parasites from Malindi, the mean *H*_*e*_ of the 8 microsatellite loci around NYD and NFD to YYD was compared. The other haplotypes could not be compared because the numbers of samples with specific haplotypes per site were small or missing. There was a reduction in the mean *H*_*e*_ around the NYD compared to NFD in Kisumu and Kericho (Table 2). The difference reached statistical difference in Kericho [*H*_*e*_ 0.667 (NYD) vs. 0.810 (NFD); *P* = 0.0097]. To the contrary, there was a reduction in mean *H*_*e*_ around the NFD compared to NYD in Kisii but did not reach statistical difference [*P* = 0.103]. When the mean *H*_*e*_ of the 8 microsatellite loci around NYD and NFD to YYD in Malindi samples were compared, data revealed there was a reduction in the mean *H*_*e*_ in YYD compared to NYD [7.3%] and NFD [7.6%], but did not reach statistical difference [YYD vs. NYD (*P* = 0.127) and YYD vs. NFD (*P* = 0.087)].

**Table 2:** Expected heterozygosity (*H*_*e*_) of main haplotypes per field site

	NYD	NFD	YYD
Kisumu	0.860	0.863	–
Kericho	0.667	0.810	–
Kisii	0.853	0.790	–
Malindi	0.780	0.783	0.707

The mean genetic differentiation index between the three Pfmdr1 haplotypes groups per each study site measured using the closest 8 microsatellite loci (± 9 kb) around the *pfmdr1*gene

Pairwise F*st* values measuring the genetic differentiation and the degree of resemblance among parasites circulating were compared for the different sites. With the exception of Kericho and Malindi which isolates had F*st* value of less than zero which is indicative of the absence of genetic differentiation, the rest had F*st* values greater than zero (three with significant P-values), indicative of genetic divergence (Table 3). Further, to investigate the relationship between increased geographic distance and the degree of resemblance, an F*st* value of 0.026 was obtained when the western Kenya sites (n = 100) were compared to the coastal Kenya (n = 32), which is over 800 km apart. Further, parasites were compared based on the different haplotypes populations (regardless of the location where they were collected) based on the pattern of SNPs in the *pfmdr1* N86Y, Y184F and D1246Y in each sample, and the genetic differentiation assessed. Of the 10 different haplotypes compared, seven gave positive F*st* values indicative of genetic divergence, with five of the seven reaching statistical difference (Table 4). The negative F*st* values are indicative of the absence of genetic differentiation, which some values could be biased due to the low sample numbers.

**Table 3:** Comparison of diversity between sites

Population 1 (n)	Population 2 (n)	F*st*	P-value
Kericho (17)	Kisumu (46)	0.048	0.001
Kisumu (46)	Malindi (32)	0.029	0.001
Kisumu (46)	Kisii (37)	0.015	0.010
Kericho (17)	Kisii (37)	0.011	0.083
Kisii (37)	Malindi (32)	0.004	0.200
Kericho (17)	Malindi (32)	− 0.017	0.453

**Table 4:** Comparison of diversity between haplotypes

Population 1 (n)	Population 2 (n)	F*st*	P-value
NFD (59)	NYD (51)	0.013	0.001
NFD (59)	YYD (13)	0.032	0.001
NYD (51)	YYD (13)	0.038	0.001
NFD (59)	YYY (4)	0.055	0.002
NYD (51)	YYY (4)	0.049	0.002
NYY (3)	YYY (4)	0.030	0.289
NYY (3)	YYD (13)	0.004	0.406
NYD (51)	NYY (3)	− 0.009	0.409
YYD (13)	YYY (4)	− 0.002	0.437
NFD (59)	NYY (3)	− 0.001	0.444

F*st* values of the parasite isolates were compared based on the haplotypes regardless of the location where the parasites were collected

The F*st* values for pairwise comparison of linked loci showed highest diversity among upstream than downstream loci. The divergence in Locus 3.6 reached statistical significance for all the sites except Kericho versus Kisumu. This was followed by loci 0, 0.16, then 0.45 upstream and − 4.2 then − 3.3 downstream (Table 5). Notably, samples from Malindi significantly differed from the rest of the study sites (*P* < 0.05) followed by Kisii. Additional analysis summarizing the F*st* for haplotypes within each site against haplotypes at other sites is shown in Additional file 1.

**Table 5:** Comparison of diversity between sites for each locus

Locus	Population 1 (n)	Population 2 (n)	F*st*	P-values
− 9.3	Kericho (17)	Kisumu (46)	0.003	0.275
	Kericho (17)	Kisii (37)	0.013	0.122
	Kisumu (46)	Kisii (37)	0.004	0.197
	Kericho (17)	Malindi (32)	0.006	0.262
	Kisumu (46)	Malindi (32)	0.000	0.448
	Kisii (37)	Malindi (32)	0.000	0.387
− 4.2	Kericho (17)	Kisumu (46)	0.010	0.096
	Kericho (17)	Kisii (37)	0.001	0.417
	Kisumu (46)	Kisii (37)	0.000	0.418
	Kericho (17)	Malindi (32)	0.034	0.007
	Kisumu (46)	Malindi (32)	0.010	0.047
	Kisii (37)	Malindi (32)	0.020	0.015
− 3.3	Kericho (17)	Kisumu (46)	0.013	0.076
	Kericho (17)	Kisii (37)	0.000	0.433
	Kisumu (46)	Kisii (37)	0.000	0.420
	Kericho (17)	Malindi (32)	0.007	0.222
	Kisumu (46)	Malindi (32)	0.047	0.001
	Kisii (37)	Malindi (32)	0.031	0.001
0	Kericho (17)	Kisumu (46)	0.016	0.094
	Kericho (17)	Kisii (37)	0.000	0.394
	Kisumu (46)	Kisii (37)	0.039	0.001
	Kericho (17)	Malindi (32)	0.035	0.024
	Kisumu (46)	Malindi (32)	0.056	0.001
	Kisii (37)	Malindi (32)	0.099	0.001
0.16	Kericho (17)	Kisumu (46)	0.020	0.030
	Kericho (17)	Kisii (37)	0.040	0.003
	Kisumu (46)	Kisii (37)	0.000	0.442
	Kericho (17)	Malindi (32)	0.034	0.009
	Kisumu (46)	Malindi (32)	0.009	0.068
	Kisii (37)	Malindi (32)	0.044	0.001
0.45	Kericho (17)	Kisumu (46)	0.048	0.001
	Kericho (17)	Kisii (37)	0.011	0.083
	Kisumu (46)	Kisii (37)	0.015	0.010
	Kericho (17)	Malindi (32)	0.000	0.453
	Kisumu (46)	Malindi (32)	0.029	0.001
	Kisii (37)	Malindi (32)	0.004	0.200
3.6	Kericho (17)	Kisumu (46)	0.013	0.061
	Kericho (17)	Kisii (37)	0.074	0.001
	Kisumu (46)	Kisii (37)	0.023	0.005
	Kericho (17)	Malindi (32)	0.018	0.043
	Kisumu (46)	Malindi (32)	0.016	0.007
	Kisii (37)	Malindi (32)	0.036	0.001
9.1	Kericho (17)	Kisumu (46)	0.000	0.407
	Kericho (17)	Kisii (37)	0.003	0.304
	Kisumu (46)	Kisii (37)	0.000	0.362
	Kericho (17)	Malindi (32)	0.000	0.423
	Kisumu (46)	Malindi (32)	0.000	0.415
	Kisii (37)	Malindi (32)	0.000	0.381

### Drug-resistant alleles genetic lineages

**Fig. 3:**
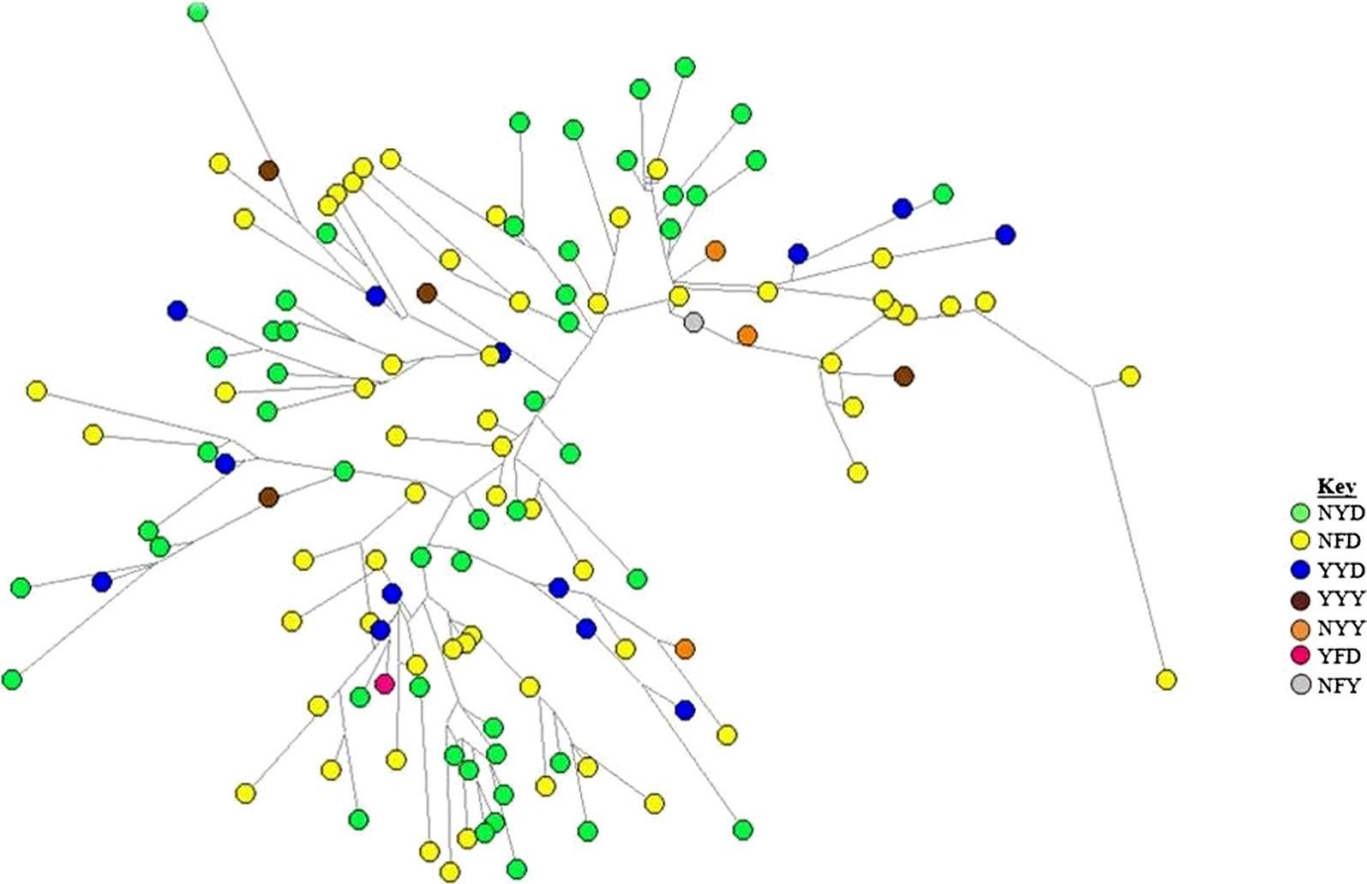
Median-joining network diagram showing genetic lineages of *pfmdr1* alleles in Kenya. The multilocus microsatellite haplotype profiles (using − 9.3, − 4.2, − 3.3, 0, 0.16, 0.45, 3.6, and 9.1 kb flanking *pfmdr1* gene) were constructed to generate networks. A total of 132 *Plasmodium falciparum* isolates were analysed and gave a total of 132 unique 8-loci microsatellites haplotypes. For allele sizes and other details please refer to Additional file 4. Each circle in the network represents a unique microsatellite haplotype colour indicating the *pfmdr1* haplotype associated (see key for the colour codes)

The 8 closest microsatellite loci (from the 13 that were typed) around the gene (9.3–9.1 kb) were used to construct multilocus haplotypes for all the isolates. The use of the 8 closest loci allowed for a more comprehensive comparison ourdata with data obtained in studies conducted in Ghana and Cambodia [44, 52]. At the 8 loci, there was no matching haplotype; the 132 isolates classified into 132 different haplotypes which is consistent with multiple independent lineages of *pfmdr1* alleles (Fig. 3). Interestingly however, there were three pairs of isolates which had all but two microsatellite loci matching (Table 6). In the three sets, each set had the matching *pfmdr1* haplotype alleles with two of those sets (NYD and YYD) from Malindi (Additional files 2, 3).

**Table 6:** List of sample pairs matching at all but 2 loci

Sample	Pop	− 9.3	− 4.2	**− **3.3	0	0.16	0.45	3.6	9.1
Kericho 6	NFD	150	156	94	196	216	*156*	176	*276*
Kisumu 27	NFD	150	156	94	196	216	*180*	176	*284*
Malindi 8	NYD	148	*202*	124	196	234	156	188	*290*
Malindi 13	NYD	148	*200*	124	196	234	156	188	*276*
Malindi 6	YYD	*148*	202	128	*192*	236	144	168	280
Malindi 27	YYD	*150*	202	128	*148*	236	144	168	280

The mismatched microsatellite loci are shown in italic. The numerical value indicate the length of each microsatellite

## Discussion

The data in this study shows differential site and region specific prevalence of SNPs associated with drug resistance in the *pfmdr1* gene. The overall prevalence of *pfmdr1* N86, 184F and D1246 were 86.4%, 47.5% and 93.9%, respectively. However, when analysed based on the region comparing western Kenya (Kisumu, Kericho and Kisii) vs. coastal Kenya (Malindi), the prevalence of *pfmdr1* N86, 184F and D1246 was 92.9% vs. 66.7%; 53.5% vs. 24.2%; 96% vs. 87.9%, respectively. The *pfmdr1* N86, 184F and D1246 genotypes are associated with AL selection [19–22]. Ingasia et al. [53] recently showed that parasites from western Kenya have high parasite genetic diversity compared to those from the coastal region of Kenya. This coincides with the reports of reduction of malaria infections and transmission in the coastal region [54–57]. The high prevalence of N86, 184F and D1246 genotypes in western Kenya compared to coastal Kenya is consistent with AL selection.

Haplotype analysis have shown lumefantrine susceptibility decreases in the order of NFD, NYD, YYY and YYD [27], with parasites gradually acquiring tolerance, starting with N86, followed by the combination of N86 + D1246 and thereafter, the combination of N86 + 184F + D1246. This observation has been corroborated by field studies [22, 24, 58, 59]. Similarly, in this study, NFD haplotype was the most prevalent haplotype followed by NYD and then YYD, depicting the role of lumefantrine drug pressure in the Kenyan parasite population. When analysed per region, western Kenya had NFD and NYD prevalence of 51% and 39%, respectively, compared to the coastal region which was 25% and 37.5%, respectively. A previous study that analysed samples collected in 2012/13 from coastal region showed NFD and NYD at a prevalence of 31.9% and 66%, respectively [34]. This data suggests that there might be less AL selection pressure in parasites in coastal region of Kenya compared to western region of Kenya.

Soft sweeps are selective events in which there have been multiple origins of the beneficial alleles [60–62]. Soft sweeps have variation in markers flanking selected alleles with multiple origins when mutations are high and populations are large [47, 60–62]. The significantly low mean *H*_*e*_ surrounding *pfmdr1* compared to the mean *H*_*e*_ at the neutral loci imply that the gene has undergone selection in Kenya. The reduction of mean *H*_*e*_ around mutant alleles compared to the respective wild type alleles is an indication of positive directional selection. Analysis of data from the four field sites indicated there was no statistical difference in mean *H*_*e*_ between NYD and NFD haplotypes. However, there was a statistical significant reduction of mean *H*_*e*_ surrounding YYD compared to NYD. When compared per study site, each site indicated unique selection pressure. In Kisumu, there was no difference in mean *H*_*e*_ between NYD and NFD, whereas in Kericho there was a statistical significant reduction of mean *H*_*e*_ surrounding NYD compared to NFD. In Kisii, the selection pressure was the opposite of what was seen in Kericho and in Malindi, reduction was only present in the mean *H*_*e*_ surrounding YYD compared to either NYD or NFD.

Multiple independent lineages of *pfmdr1* allele have been previously described for parasites in Ghana [52] and Cambodia [38]. Similarly, the current study demonstrated the presence of independent genetic lineages for all the *pfmdr1* alleles. Interesting however, for the study that described parasites in Ghana [52], the authors observed an increase in linkage disequilibrium among loci around YFD haplotype, which suggested one major and a few minor lineages of this haplotype. Only one sample with the YFD genotype was observed in current study.

F*st* statistics analyses among the linked loci showed geographic distance between the field sites, and appear to play a role in selection. This was evident when western Kenyan parasites were compared to coastal Kenyan parasites, which are geographically separated by more than 800 km. The significant difference in F*st* between samples from the two separate locales agree with previous studies which have showed greater genetic distance between physically isolated populations [56]. There was however evidence of marginal genetic sharing among these populations that could be partly due to dispersal of parasites across these regions [63]. Parasites from Kericho and Malindi were exception because they were identical despite of the distance between the two sites. Loci − 9.3 and 9.1 were comparable across all populations depicting minimal involvement in selection.

Drug pressure has been implicated as a key driver of selection [64]. The samples clustered into seven haplotypes of the *pfmdr1* N86Y, Y184F and D1246Y showed F*st* values greater than zero suggesting increasing divergence among most haplotypes. Chloroquine use before the year 2000 was shown to be the greatest force behind selection in these loci. Since chloroquine withdrawal more than 20 years ago, there has been return of wild-type at *pfmdr1* 86 and 1246, but emerging *pfmdr1* 184F [31], which is associated with lumefantrine selection [65]. These findings show divergence which appear to suggest different lumefantrine pressure in the different field sites, or presence of other factors that influence selection differently.

Since switching of the first-line, anti-malarial against uncomplicated malaria in Kenya from chloroquine to SP, and then to AL in 2006 [15, 17], studies have shown trends of recovery of chloroquine sensitive parasites [31, 33, 34]. These trends have been shown in other African countries as well [35, 66–72]. However, the rates with which the changes occur are different from one region to another, or one country to another. This is the first study which directly compares the prevalence of *pfmdr1* alleles and genetic lineages in samples from the western Kenya to those in the coastal Kenya. Notably, the populations are structured, with those from coastal region showing significant variation in loci surrounding the allele under selection compared to those from the western Kenya loci. Study by O’Meara et al. underscored declining malaria incidence in this region [73] which is attributed to intensified intervention [16]. On the contrary, there are reports of sustained malaria transmission in western Kenya [16, 74] despite similar country-wide transitions of interventions including in drug treatment policy [75, 76]. Findings in this study which show significant variations between these populations provide evidence for differential selection pressure between the different malaria transmission regions of Kenya, especially the western region of Kenya compared to the coastal region. Indeed, a recent study showed western Kenya parasites have high genetic diversity compared to those in coastal Kenya [53]. The difference in selection pressure can be attributed to disease prevalence, genetic diversity of the parasite population, anti-malarial drug usage and cultural behaviour of the different patient populations alongside environmental factors that modulate vector density [77]. Adherence to anti-malarial drug treatment is a challenge as evident by a recent case report of attenuated-responsiveness to AL treatment in western Kenya [78].

## Conclusions

The study shows different prevalence of *pfmdr1* alleles in different regions of Kenya, especially western Kenya compared to coastal Kenya. Further, evidence of soft sweeps in *pfmdr1* has been shown, but the direction of the selection for the *pfmdr1* haplotypes is different from one region to another, which can be explained by factors such as difference in parasite genetic diversity, drug pressure and much more. This finding poses further challenges for malaria control programmes in malaria endemic countries because transmission rates might change differently in the same country, which might require different malaria control strategies. It would be worthwhile to use this type of data as an additional molecular surveillance tool for guiding decisions for effective malaria control policies based on the region and not at a country wide level.

## Additional files

**Additional file 1.**The additional material table with microsatellites data of 8 closest loci flanking the *Pfmdr1* gene, and the corresponding SNP haplotypes data for the samples from the four different sites.

**Additional file 2.** The expected heterozygosity (*H*_*e*_) at microsatellite loci flanking *Pfmdr1* alleles. The dashed line crossing the y-axis indicates the mean *H*_*e*_ at 7 neutral microsatellite loci on chromosome 2 and 3. The other lines indicate other Pfmdr1 alleles NFD (n = 59), NYD (n = 51), YYD (n = 13), YYY (n = 4), and NYY (n = 3).

**Additional file 3.** The expected heterozygosity (*H*_*e*_) at microsatellite loci flanking Pfmdr1 alleles. The dashed line crossing the y-axis indicates the mean *H*_*e*_ at 7 neutral microsatellite loci on chromosome 2 and 3. The other lines indicate other *Pfmdr1* alleles NFD (n = 59), NYD (n = 51), and YYD (n = 13).

**Additional file 4.** Represents diversity (F*st*) of haplotypes across all sites.
