# Supplementary material for: Selective sweeps and genetic lineages of Plasmodium falciparum multi-drug resistance (pfmdr1) gene in Kenya
Source: Malar J. 2018 Oct 30;17:398. doi: 10.1186/s12936-018-2534-8 (PMC6208105; doi:10.1186/s12936-018-2534-8)
Supplement: Supplementary file 1 — Additional file 1.The additional material table with microsatellites data of 8 closest loci flanking the Pfmdr1 gene, and the corresponding SNP haplotypes data for the samples from the four different sites. [file 12936_2018_2534_MOESM1_ESM.pdf]

Data from eight microsatellites flanking the pfmdr1 gene

Sample Name	-9.3	-4.2	-3.3	0	0.16	0.45	3.6	9.1	*Pfmdr1* SNP Haplotypes
3D7	166	198	122	194	220	190	180	286	NYD
Kericho 1	148	184	?	196	230	188	172	280	NFD
Kericho 2	148	204	124	194	216	152	174	282	NYD
Kericho 3	148	196	134	194	236	156	184	286	NFD
Kericho 4	150	194	92	192	236	184	170	286	NFD
Kericho 5	150	156	92	196	220	156	174	280	NFD
Kericho 6	150	156	94	196	216	156	176	276	NFD
Kericho 7	148	198	132	194	218	180	174	286	NYD
Kericho 8	152	156	94	192	236	156	176	282	NFD
Kericho 9	154	194	120	150	220	170	162	284	NYD
Kericho 10	156	190	116	212	222	178	126	252	NFD
Kericho 11	150	184	128	198	216	164	184	308	NFD
Kericho 12	152	194	128	192	232	190	170	284	NFD
Kericho 13	138	200	130	196	236	146	162	280	NFD
Kericho 14	148	204	116	194	218	164	160	254	NYD
Kericho 15	148	194	120	198	220	178	176	284	NYD
Kericho 16	160	184	94	194	220	152	176	254	NYD
Kericho 17	150	202	96	190	218	160	162	288	NFD
Kisumu 1	148	192	126	192	232	144	210	280	NFD
Kisumu 2	148	204	122	190	214	150	130	284	NYD

Kisumu 3	146	190	90	206	222	180	168	266	NFD
Kisumu 4	148	188	126	196	220	154	142	252	NYD
Kisumu 5	126	188	122	190	224	176	146	282	NFD
Kisumu 6	150	190	?	190	220	176	172	284	NFD
Kisumu 7	150	200	120	192	248	150	170	244	NFD
Kisumu 8	146	200	114	154	214	150	162	280	NFD
Kisumu 9	156	188	?	186	246	172	174	278	NYD
Kisumu 10	162	182	90	176	234	172	174	256	NYD
Kisumu 11	166	194	122	196	216	152	176	280	NFD
Kisumu 12	140	194	?	192	224	182	158	288	NYD
Kisumu 13	156	156	122	194	242	158	166	250	NFD
Kisumu 14	156	192	120	176	236	176	166	280	NFD
Kisumu 15	152	150	130	154	236	174	146	252	NFD
Kisumu 16	160	206	124	154	230	150	194	280	NYD
Kisumu 17	144	190	130	192	220	190	172	278	NFD
Kisumu 18	160	198	120	196	218	152	172	286	NYD
Kisumu 19	148	200	124	192	224	206	162	280	NYD
Kisumu 20	150	198	94	196	218	148	172	284	NFD
Kisumu 21	150	156	94	192	216	190	170	280	NFD
Kisumu 22	150	196	126	190	232	164	170	254	NFD
Kisumu 23	148	196	126	190	214	158	170	284	NFD
Kisumu 24	156	192	114	190	216	176	182	282	NYD
Kisumu 25	150	196	90	192	246	152	174	280	NFD
Kisumu 26	148	190	?	186	228	182	182	280	NFD
Kisumu 27	150	156	94	196	216	180	176	284	NFD

Kisumu 28	154	196	94	196	226	176	174	280	NYD
Kisumu 29	158	188	120	176	224	176	176	282	NFD
Kisumu 30	152	190	126	176	238	188	182	284	NYD
Kisumu 31	150	204	128	194	216	176	166	280	NFD
Kisumu 32	?	194	114	194	216	176	158	246	YYD
Kisumu 33	156	202	122	192	232	152	182	278	NYD
Kisumu 34	?	186	90	154	236	170	?	282	YYD
Kisumu 35	156	210	128	192	214	176	188	280	NFD
Kisumu 36	158	200	122	192	232	186	192	276	YYD
Kisumu 37	148	190	120	202	226	184	166	280	NYD
Kisumu 38	154	208	118	186	242	186	170	280	NFD
Kisumu 39	158	200	122	194	236	146	166	252	NYY
Kisumu 40	146	204	132	148	226	186	178	280	NFD
Kisumu 41	148	196	118	148	230	?	148	246	NYD
Kisumu 42	164	214	116	194	226	148	168	288	NFD
Kisumu 43	?	182	94	192	220	176	174	282	NFY
Kisumu 44	150	198	92	194	214	176	176	288	NYY
Kisumu 45	144	204	122	192	214	180	172	284	NFD
Kisumu 46	156	194	126	192	214	180	172	282	NYD
Kisii 1	148	186	?	194	222	180	158	256	NYD
Kisii 2	150	204	116	192	218	166	172	278	NFD
Kisii 3	146	192	132	190	234	164	174	280	NYD
Kisii 4	148	156	94	198	230	196	176	280	NYD
Kisii 5	?	152	114	194	220	176	172	280	YYY
Kisii 6	158	188	122	194	210	174	154	252	NYD

Kisii 7	150	192	126	190	232	158	172	284	NFD
Kisii 8	?	194	?	174	214	180	172	282	YYD
Kisii 9	146	206	128	192	214	176	172	282	NFD
Kisii 10	148	196	76	194	226	184	172	286	NYD
Kisii 11	148	186	94	194	238	180	176	308	NYD
Kisii 12	150	194	116	192	216	178	180	280	NFD
Kisii 13	150	194	118	174	214	180	172	284	NFD
Kisii 14	158	196	124	192	240	158	170	278	NYD
Kisii 15	148	194	122	192	218	172	166	278	NFD
Kisii 16	150	206	124	194	218	146	170	282	NYD
Kisii 17	150	200	94	194	228	178	186	282	NYD
Kisii 18	148	156	94	194	226	156	172	284	NFD
Kisii 19	?	198	114	194	214	180	172	280	YYD
Kisii 20	146	196	116	200	226	156	186	280	NYD
Kisii 21	148	194	?	184	214	152	172	282	NFD
Kisii 22	150	188	154	204	222	186	172	284	NFD
Kisii 23	168	194	?	194	220	176	172	284	NFD
Kisii 24	162	194	118	174	220	154	182	282	NYD
Kisii 25	138	202	112	192	220	156	174	280	NFD
Kisii 26	168	196	120	194	232	180	182	280	NFD
Kisii 27	168	192	120	196	232	152	182	282	NFD
Kisii 28	154	190	88	196	214	148	170	280	NYD
Kisii 29	148	208	120	196	228	180	160	282	NYD
Kisii 30	154	194	118	174	224	154	?	282	NYD
Kisii 31	?	202	114	190	214	148	168	280	YFD

Kisii 32	146	198	126	194	222	166	172	280	NYD
Kisii 33	168	202	114	194	236	174	164	288	NFD
Kisii 34	144	196	126	192	214	152	172	276	NFD
Kisii 35	146	198	130	194	228	180	168	276	NYD
Kisii 36	158	190	94	192	234	196	174	276	NYD
Kisii 37	150	190	118	188	220	190	190	244	NYD
Malindi 1	152	188	90	192	220	152	184	280	NFD
Malindi 2	150	204	128	176	230	144	168	278	YYD
Malindi 3	146	202	90	178	234	156	186	280	NYD
Malindi 4	146	182	94	196	216	178	176	280	NYY
Malindi 5	150	202	90	196	224	188	186	284	NYD
Malindi 6	148	202	128	192	236	144	168	280	YYD
Malindi 7	?	198	126	174	216	152	140	252	YYD
Malindi 8	148	202	124	196	234	156	188	290	NYD
Malindi 9	154	196	116	196	216	148	170	278	YYD
Malindi 10	150	196	110	196	232	160	168	276	NFD
Malindi 11	148	184	128	178	232	162	172	284	NFD
Malindi 12	?	198	116	196	222	154	162	250	YYY
Malindi 13	148	200	124	196	234	156	188	276	NYD
Malindi 14	152	196	116	196	216	152	174	282	YYD
Malindi 15	150	194	122	194	218	176	178	268	NYD
Malindi 16	148	184	124	194	216	170	162	286	NFD
Malindi 17	150	190	?	192	224	144	174	282	NFD
Malindi 18	158	206	?	196	224	156	186	282	NYD
Malindi 19	148	202	116	196	238	164	186	280	NYD

Malindi 20	150	198	116	148	214	152	178	248	YYY
Malindi 21	?	198	116	148	216	180	176	282	YYD
Malindi 22	?	198	126	174	222	180	160	280	YYY
Malindi 23	158	200	132	174	228	168	180	280	NYD
Malindi 24	148	190	116	148	184	186	172	284	NFD
Malindi 25	156	192	116	148	226	158	172	284	NFD
Malindi 26	150	200	94	198	234	186	172	286	NYD
Malindi 27	150	202	128	148	236	144	168	280	YYD
Malindi 28	154	198	130	188	180	160	138	?	NYD
Malindi 29	146	200	116	192	216	146	178	280	NFD
Malindi 30	146	190	134	196	224	156	186	280	NYD
Malindi 31	?	200	120	178	216	180	176	246	NYD
Malindi 32	?	196	116	178	216	178	176	252	YYD
