# Supplementary figures and images for: Selective sweeps and genetic lineages of Plasmodium falciparum multi-drug resistance (pfmdr1) gene in Kenya
Source: Malar J. 2018 Oct 30;17:398. doi: 10.1186/s12936-018-2534-8 (PMC6208105; doi:10.1186/s12936-018-2534-8)

Comparison of  $H_e$  between alleles in *Pfmdr1* gene

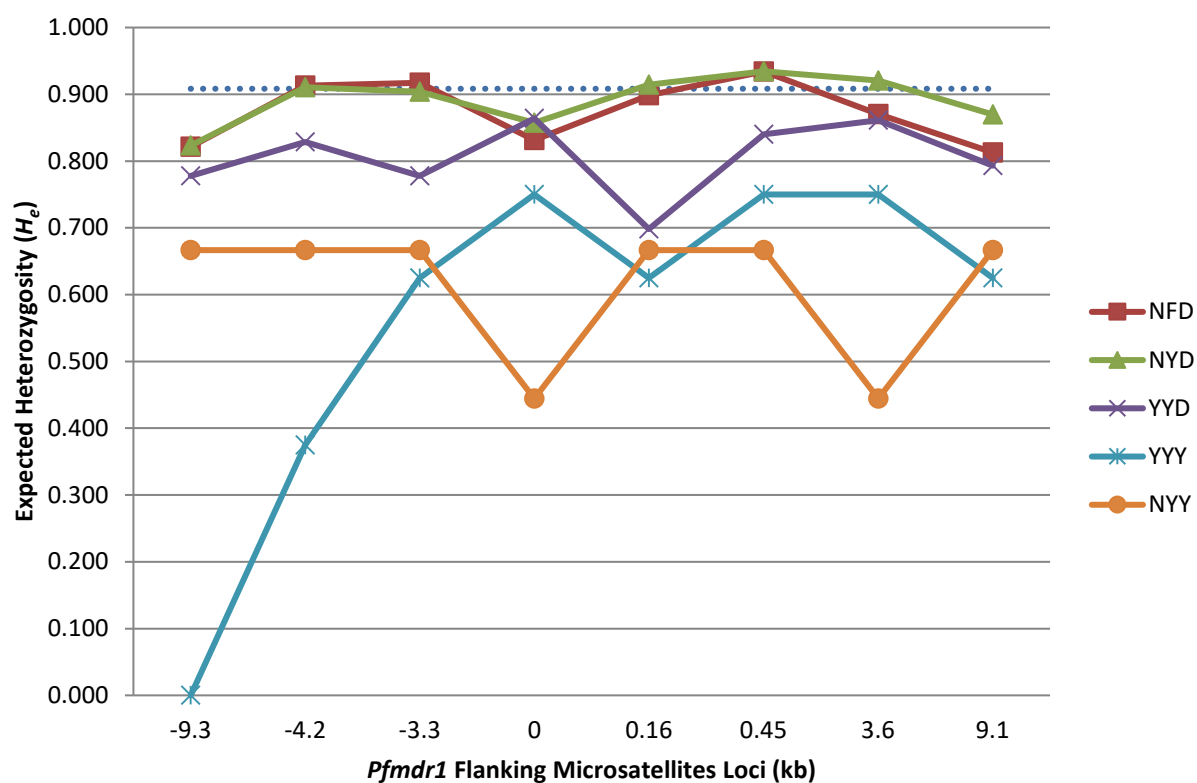

Supplement: Supplementary file 2 — Additional file 2. The expected heterozygosity (He) at microsatellite loci flanking Pfmdr1 alleles. The dashed line crossing the y-axis indicates the mean He at 7 neutral microsatellite loci on chromosome 2 and 3. The other lines indicate other Pfmdr1 alleles NFD (n = 59), NYD (n = 51), YYD (n = 13), YYY (n = 4), and NYY (n = 3). [file 12936_2018_2534_MOESM2_ESM.pdf]

Comparison of  $H_e$  between alleles in *Pfmdr1* gene

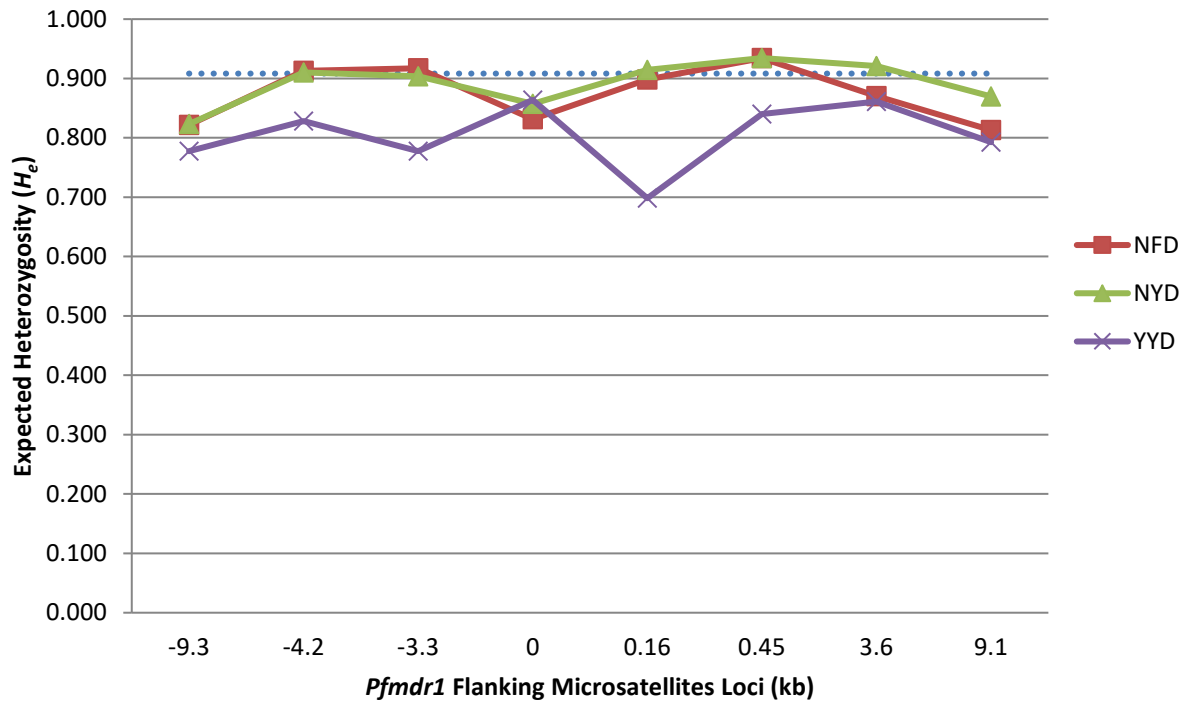

Supplement: Supplementary file 3 — Additional file 3. The expected heterozygosity (He) at microsatellite loci flanking Pfmdr1 alleles. The dashed line crossing the y-axis indicates the mean He at 7 neutral microsatellite loci on chromosome 2 and 3. The other lines indicate other Pfmdr1 alleles NFD (n = 59), NYD (n = 51), and YYD (n = 13). [file 12936_2018_2534_MOESM3_ESM.pdf]
