# Supplementary material for: Selective sweeps and genetic lineages of Plasmodium falciparum multi-drug resistance (pfmdr1) gene in Kenya
Source: Malar J. 2018 Oct 30;17:398. doi: 10.1186/s12936-018-2534-8 (PMC6208105; doi:10.1186/s12936-018-2534-8)
Supplement: Supplementary file 4 — Additional file 4. Represents diversity (Fst) of haplotypes across all sites. [file 12936_2018_2534_MOESM4_ESM.pdf]

Comparison of F*st* within each site against haplotypes

Population 1 (n)	Population 2 (n)	Fst	P Value
KCONYD (6)	MDHNYD (12)	0.106	0.001
KDHNFD (25)	KCONYD (6)	0.074	0.001
KDHNFD (25)	MDHNYD (12)	0.047	0.001
KDHNFD (25)	MDHYYD (8)	0.053	0.001
KDHNYD (15)	MDHYYD (8)	0.063	0.001
KSINFD (15)	MDHNYD (12)	0.077	0.001
KSINFD (15)	MDHYYD (8)	0.096	0.001
KSINYD (18)	MDHYYD (8)	0.073	0.001
MDHNYD (12)	MDHYYD (8)	0.094	0.001
KCONYD (6)	MDHYYD (8)	0.123	0.002
KDHNYD (15)	MDHNYD (12)	0.039	0.002
KSINFD (15)	KSINYD (18)	0.032	0.002
KSINFD (15)	MDHYYY (3)	0.119	0.002
MDHNYD (12)	KSIYYD (2)	0.158	0.002
KDHNYD (15)	MDHYYY (3)	0.081	0.003
KSINFD (15)	KCONYD (6)	0.067	0.003
KCONFD (11)	KSINFD (15)	0.034	0.004
KCONFD (11)	KCONYD (6)	0.075	0.004
KCONYD (6)	KDHNYD (15)	0.048	0.004
KDHNFD (25)	MDHYYY (3)	0.081	0.004
KCONFD (11)	KSIYYD (2)	0.161	0.007
KCONYD (6)	MDHYYY (3)	0.161	0.007

KCONFD (11)	MDHYYY (3)	0.1	0.009
KDHNFD (25)	KSIYYD (2)	0.1	0.009
KSINYD (18)	MDHYYY (3)	0.076	0.01
MDHNYD (12)	MDHYYY (3)	0.106	0.011
KCONYD (6)	KSIYYD (2)	0.2	0.012
KCONFD (11)	KDHNYD (15)	0.026	0.013
KSINYD (18)	MDHNYD (12)	0.028	0.013
MDHNFD (8)	MDHNYD (12)	0.039	0.014
KDHNFD (6)	KSINYD (18)	0.012	0.015
KSIYYD (2)	MDHYYD (8)	0.176	0.015
KCONFD (11)	KSINYD (18)	0.024	0.016
KCONFD (11)	MDHYYD (8)	0.047	0.017
KDHNYD (15)	KSIYYD (2)	0.098	0.017
MDHNFD (8)	KSIYYD (2)	0.131	0.025
KCONFD (11)	MDHNYD (12)	0.026	0.026
KDHNFD (25)	KSINFD (15)	0.014	0.027
KSINFD (15)	KDHNYD (15)	0.016	0.037
KCONYD (6)	KSINYD (18)	0.028	0.049
MDHNFD (8)	MDHYYY (3)	0.078	0.051
KDHNYD (15)	KSINYD (18)	0.012	0.053
MDHNFD (8)	KCONYD (6)	0.045	0.056
KSINYD (18)	KSIYYD (2)	0.07	0.079
MDHNFD (8)	KSINYD (18)	0.017	0.081
KDHNYY (2)	MDHYYD (8)	0.104	0.091
KCONYD (6)	KDHYYD (3)	0.08	0.096

MDHNFD (8)	MDHYYD (8)	0.029	0.103
KCONFD (11)	KDHNFD (25)	0.01	0.108
MDHNYD (12)	KDHNYY (2)	0.063	0.11
KSINFD (15)	KDHYYD (3)	0.036	0.114
KDHYYD (3)	MDHYYD (8)	0.057	0.116
KSINFD (15)	KDHNYY (2)	0.055	0.12
KSINFD (15)	KSIYYD (2)	0.048	0.128
MDHNYD (12)	KDHYYD (3)	0.042	0.137
KDHNYY (2)	KSIYYD (2)	0.185	0.152
KDHNYD (15)	KDHNYY (2)	0.044	0.156
MDHYYD (8)	MDHYYY (3)	0.051	0.175
MDHNFD (8)	KDHNYY (2)	0.062	0.197
KDHYYD (3)	MDHYYY (3)	0.096	0.209
KCONFD (11)	KDHYYD (3)	0.029	0.218
KCONYD (6)	KDHNYY (2)	0.071	0.219
KDHNFD (25)	KDHYYD (3)	0.013	0.243
KDHNYY (2)	MDHYYY (3)	0.109	0.252
KSIYYD (2)	MDHYYY (3)	0.103	0.264
KSINYD (18)	KDHYYD (3)	0.012	0.288
KCONFD (11)	KDHNYY(2)	0.01	0.318
KDHNFD (25)	KDHNYY (2)	0.003	0.331
KDHNYD (15)	KDHYYD (3)	0.008	0.348
KDHYYD (3)	KSIYYD (2)	0.062	0.349
KSINYD (18)	KDHNYY (2)	-0.005	0.361
KSINFD (15)	MDHNFD (8)	-0.005	0.445

MDHNFD (8)	KDHYYD (3)	0.004	0.46
KCONFD (11)	MDHNFD (8)	-0.004	0.47
MDHNFD (8)	KDHNYD (15)	-0.009	0.477
KDHNFD (25)	MDHNFD (8)	-0.006	0.486
KDHNFD (25)	KDHNYD (15)	-0.004	0.491
KDHNYY (2)	KDHYYD (3)	-0.084	0.561
